# From India—International Aortic Summit: There Is Nothing Impossible for Those Who Will Try!

**DOI:** 10.1055/s-0042-1756672

**Published:** 2022-12-15

**Authors:** Mohammed Idhrees, Mohamad Bashir, Bashi V. Velayudhan

**Affiliations:** 1 Institute of Cardiac and Aortic Disorders, SRM Institutes for Medical Science (SIMS Hospitals), Chennai, Tamil Nadu, India; 2 Department of Neurovascular Research Laboratory, Faculty of Life Sciences and Education, University of South Wales, Wales, United Kingdom; 3 Department of Vascular and Endovascular Surgery, Velindre University National Health Service Trust, Health Education, and Improvement Wales (HEIW), Cardiff, United Kingdom

The aorta is a dynamic living structure, central to all vital organs with its aortovascular branches. At the center of the affair, the aorta is afflicted with aneurysm disease, a complex condition that warrants attention and a multidisciplinary huddle to understand and ameliorate its dire effects. The International Aortic Summit in Chennai, India, is not just a conference, but a confluence of multidisciplinary specialties and teams joined around a rounded table of innovation and collaboration.

The 9th International Aortic Summit 2020 (IAS2020) was a witness to such confluence and was not hindered by the untoward effects of the pandemic. We streamed live globally bringing esteemed colleagues to deliver an eye and reflective practice on their experiences and expertise. Viewed globally by over 42 countries, IAS2020 was organized into structured sessions and platforms to cover all profiles of aortic disease and surgery.

Aortic surgery practice in India was a major focus during the Summit. Discussants touched the outcome and available resources. The aim was to incentivize practicing surgeons and young aspiring colleagues to form a solid practice and surgical approach structured, devised, and based on clinical evidence and research.

Sessions dedicated to pioneers in aortic surgery were a mere reflection of sheer and driven practices. Those were imperative to impose the way the future is evolving and to incite young surgeons to follow pursuit.

Keynote speakers including Drs Joseph Coselli and Joseph Bavaria delivered their shrewd expert opinions on aortic surgery over their decades of experience; the key points and sessions are listed in Tables 1 and 2 . There were discussions on a wide range of topics related to thoracic and abdominal aortic aneurysms, aortic dissections, genetics pertaining to the aorta, interventional aortic radiology, and novel endovascular aortovascular innovations. The summit was a relishing opportunity to the current trend of aortic surgery in India and advances made across the years, best told by B.V.V., the Summit Director and propeller of innovation in aortic surgery management in India.

**Table 1:** Salient features of keynote lectures

*Prof. Joseph Coselli, Houston, TX* *Thoracic Aortic Aneurysm Surgery: Don't Quit:*
Cardiac surgical devices and prostheses have undergone multiple innovations and developments in the last few decades.
To work with our colleagues closely–cardiologists, radiologists, and vascular surgeons.
Enumerated the developments in aortic arch surgery over the years.
*Prof. Joseph Bavaria, Philadelphia, PA* *Type A Dissection: From the 30/30 club disaster to an upcoming Innovative decade*
*Most important concept in future is in regard to* “ *distal aorta* ”:
Index proximal operations will be driven and conceived by the availability of new technology endografts.
Proximalization of the conduct of aortic arch operations will continue.
Index operations “tactics”' will be driven to reduce total cardiopulmonary bypass time.
Achieve better accumulation of outcomes data on the global stage.

**Table 2:** The key points of all the sessions over 2 days

Day 1	
*Aortic Surgery in Asia–I:*	
Dr. Kay-Hyun Park, South Korea Extensive thoracic aortic replacement (ascending to distal descending thoracic) without FET via a single median sternotomy	• Extensive thoracic aorta replacement is feasible via median sternotomy and transpericardial approach • Frozen elephant trunk should be reserved for desperate cases who cannot undergo two-stage operation at a short interval
Dr. Worawong Slisatkorn, Thailand Total arch replacement in octogenarian	• Total arch replacement can be performed in octogenarians with acceptable outcomes • Team expertise and close perioperative care are mandatory to improve the operative result
Dr. Saeid Hosseini, Iran Outcomes of reoperation after acute Type A aortic dissection	Challenging issues include • Whether to repair or replace the aortic root? • The effect of the thrombosed false lumen on reoperation, partial thrombosis an independent risk factor • The extent of distal operation in the index surgery
Dr. Thodur Vasudevan, New Zealand Total body floss: a concept in difficult endovascular repair and a novel case	• Unusual method of through and through wire access • Multiple tortuosities • Out of the box solutions • Wire from the apex to femoral vessels • Substernal access
Dr. George Joseph, India Total arch fenestrated endovascular repair after surgical replacement of ascending aorta	• Residual Type A dissection can be treated by endovascular total arch fenestrated repair • Short, kinked ascending aortic surgical grafts pose a challenge to arch TEVAR • Surgeons should attempt to replace as much of the ascending aorta as possible during the initial surgery and provide a landing zone for future arch TEVAR
Dr. Murali Krishnawami, India Thoracoabdominal aneurysm—total endovascular repair	• FEVAR needs careful planning and meticulous execution • Needs multiple accesses and multiple catheters, wires, and sheaths • Can be performed by experienced centers with excellent results
Dr. Arunkumar, India The current role of transesophageal echocardiography in acute aortic syndrome	• Transesophageal echocardiogram plays a significant role inside the operative room • A good adjuvant tool for open aortic repair and endovascular procedures
*Valve in Acute Aortic Dissection:*	
Dr. Malakh Shrestha, Germany Spare the aortic valve—I do it my way!	• If aortic root is dissected but the aortic valve is normal – Normal sized aortic sinus—sandwich technique – Aortic root dilated—David's procedure when possible • Short- /long-term results of the David-I procedure in Marfan's patient are excellent • Long-term survival of hospital survivors after David-I procedure in acute dissection is comparable to elective settings
Dr. Roberto Di Bartolomeo, Italy Aortic valve sparing with Valsalva graft	• Valve sparing aortic root replacement has excellent long-term results • Bologna experience: Long-term survival at 1 year is 96.7%, 5 year is 93.9%, and 10 year is 89.5%
Dr. Laurent de Kerchove, Belgium When and how I repair the aortic valve in chronic and acute aortic regurgitation	• Aortic valve repair for pure aortic insufficiency necessitates a good understanding of the underlying mechanism and a systematic repair algorithm • Optimal durability is achieved by optimization of coaptation and by annulus (± root) stabilization
Dr. Ruggero DePaulis, Italy Biological or mechanical conduits in aortic root dissection?	• Both choices are certainly defendable • Life expectancy and the possibility of valve in valve certainly is a push for larger use of bio conduits • Proper tailoring for each patient remains the main strategy
Dr. Michael Borger, Germany Management of iatrogenic Type A aortic dissection	• Iatrogenic aortic dissection is a rare but dangerous complication of cardiac surgery and cardiac catheterization • Despite elevated risk, selected patients should be operated on emergently • Early mortality is higher for the condition than patients presenting with spontaneous Type A dissection, particularly in postcardiac surgery patients
*Aortovascular Science* :	
Dr. Wael Awad, United Kingdom Risk prediction model in aortic aneurysm surgery—unmet clinical equipoise	• Thoracic aortic aneurysm (TAA) is a dangerous, deadly, and silent disease that is notoriously difficult to detect prior to complications • Early diagnosis and management remain a critical component for limiting mortality from TAA prior to dissection • Greater understanding of the genetics of these pts and their specific genetic mutations can provide personalized aortic care, tailoring surgical recommendations for each patient
Dr. Benjamin Adams, United Kingdom Does adding a root replacement in Type A aortic dissection repair provide better outcomes?	• Addition of ARR in TAAD setting does not increase mortality or postoperative complications • ARR should be considered at the time of the emergency repair, especially in young patients with a degree of root dilatation and/or aortic valve regurgitation
Dr. John Elefteriades, United States Update on genetic dictionary	• A new era of molecular identification of individuals at risk for thoracic aortic aneurysm • Personalized strategic management of these individuals
Dr. Ourania Preventza, United States Extent of repair for Type I. How long and why?	• Distal extent preferably hemiarch, unless a total arch replacement is necessary • Total arch replacement is performed when the entry tear is within the greater curvature, severe compression of the true lumen • When stented, a 10-cm antegrade delivery is preferred
Dr. Arminder Jassar, United States The changing surgical approaches for Type A dissection	• Ascending aortic replacement will suffice for many, but not all, patients with acute Type A dissection • While the goal of the operation is to have an alive patient, it is also reasonable to plan for the future • For patients with acute malperfusion, alternative strategies or adjunct techniques should be considered to ensure reperfusion
Day 2	
*Aortic Surgery in Asia – II* :	
Dr. Bashi Velayudhan, India Acute aortic syndrome (AAS) in India—What do we know?	• Possibility of a lower incidence of the acute aortic syndrome as compared to the western world (due to diabetes mellitus) • Majority of patients are in the younger age group • Last 15 years—more centers to handle AAS • Distal aorta in Marfan's syndrome–still a challenge
Dr. Shiv Choudhary, India Inexpensive way to manage the arch in acute Type A dissection	• Lack of expensive devices should not preclude lifesaving surgery in aortic dissection • It is possible to operate with successful results in a resource constrained setup
Dr. Zile Meharwal, India Raising the bar... Hemodynamics in the small aortic root	• Small aortic root poses a challenge for surgeons • Patient prosthesis mismatch (PPM) is common with small aortic root • All measures should be taken to avoid PPM
Dr. Mohammed Idhrees, India Decision-making in acute aortic dissection	• Decision-making in the management of acute aortic dissection is crucial • When Plan A is not smooth, always choose Plan B • Select the best and simplest option which suits the team
Dr. Karthikeyan, India Gated CTA of aorta–optimal imaging of aortic disease	• Imaging with CT plays a central role in diagnosis to allow expedited management • Helps for clinical risk assessment and establishing a definitive diagnosis • CT scans to be performed with aim of motion-free images • Dedicated injection protocol should be used
*TEVAR/Hybrid Aortic* :	
Dr. Heinz Jakob, Germany FET innovation	• E-vita open neo has a family of graft variations • The graft suits all kinds of aortic pathologies
Dr. Nimesh Desai, United States Sequential branched arch TEVAR	• Index arch operations will be driven and conceived by the new availability of new technology endografts
Dr. Martin Czerny, Germany Midterm results of branched endovascular aortic arch repair	• Total endovascular aortic arch is a safe and reproducible technique • Primarily for nonsurgical candidates • Pathology determines the mode of treatment • Creation of aortic centers with the entire armamentarium will aid in doing the right things in the right patients • Will reduce the need for combined vascular/endovascular procedures
Dr. Martin Grabenwoger, Austria Management of Type 1 endoleak following TEVAR	• Treatment strategy for Type I endoleak after TEVAR has to be decided on a case-by-case basis • Decision endovascular–hybrid–surgery (FET) dependent on– Anatomy of the aortic arch – Risk profile of the patient (suitable for surgery?) – Is it possible to create a proximal landing zone for an endovascular extension by aortic arch rerouting procedures? • Open surgery by the FET technique can be performed with good results when Type I endoleak cannot be treated by endovascular techniques?
Dr. Edward Chen, United States Redo-aortic arch surgery	• Redoaortic arch surgeries are high-risk and technically demanding procedures performed with acceptable morbidity and mortality • Careful operative planning and execution are paramount for optimal clinical outcome • Operative outcomes were impacted by several factors including cardiac function and surgical complexity, but not by prior aortic procedures
Dr. Maximilian Kreibich, Germany dSINE for FET and TEVAR	• dSINE may develop at any time after FET procedure and the risk of dSINE development after FET procedure is substantial • No independent predictors for the development of dSINE were identified, but Thoraflex has a stiffer distal end as compared with E-Vita • Reinterventions for dSINE were associated with a very good clinical outcome
Dr. Cherri Abraham, United States Treatment of PAU and IMH in the thoracic aorta	• Penetrating aortic ulcer and intramural hematoma are both clinically complex and part of a clinical spectrum of acute aortic syndromes • Treatment strategies include reduction in aortic wall stress and tailoring the surgical approach to the patient and lesion
*Downstream Aorta* :	
Dr. Anthony Estrera, United States Open techniques in TAAAR in aneurysm repair	• Individualized standard of approach for aneurysm patient using FLAP (fragility/life expectancy/anatomy/pathology) • Vital importance of the team effort • Open and endovascular techniques are complimentary to each other and not competitive with one another
Dr. Joseph Coselli, United States Open techniques in TAAAR in chronic dissection	• Patients with chronic dissection tend to undergo extensive thoracoabdominal repair • A variety of operative strategies are targeted to aortic dissection • When compared with patients without dissection, those with chronic dissection are inclined to get extensive repair
Dr. Leonard Girardi, United States Emergency thoracoabdominal aortic aneurysm scenario management	• Thoracoabdominal aneurysm emergencies are high-risk undertaking • Endovascular and open repair techniques performed with similar operative risk in experience centers • Open repair is associated with fewer early- /late- reinterventions
Dr. Roberto Chiesa, Italy Open thoracoabdominal following endovascular intervention	• Close follow-up is essential after TEVAR • Open TAAAR conversion is technically challenging • Surgery can be performed with acceptable results in centers with an “aortic team” • Increased mortality in cases of infection and retrograde dissection
Dr. Germano Melissano, Italy Spinal cord ischemia in open and endovascular repair	• Spinal cord ischemia prevention requires optimizing all aspects of the procedures • CSF drainage is a valid adjunct; however, it comes with several potential serious complications • The problem is still not resolved, and more research is needed

Abbreviations: ARR, aortic root replacement; CSF, cerebrospinal fluid; CTA, computed tomography angiography; dSINE, distal stent graft induced new entry; FET, frozen elephant trunk; FEVAR, fenestrated aneurysm repair; IMH, intramural hematoma; PAU, penetrating atherosclerotic ulcer; TAAD, Type A aortic dissection; TAAAR, thoracoabdominal aortic aneurysm repair; TEVAR, thoracic endovascular aortic repair;

Following on to IAS2020, we developed a strong collaboration with AORTA and reflected on the key topics selected from the core of IAS2020. The selected topics were the crux of the two consecutive days of the summit and formed this focused issue. The articles collated were debates on pertinent topics which remain controversial and require profound attention. Practical sessions highlighted during the summit were invigorated and supported by contributors including innovative surgical approaches that emerged over the past years. The endovascular side of the program was thoroughly covered adding a touchstone and face value to furthering aortic surgery in current and future eras. The thoracoabdominal part was again a station that intrigued the audience during the Summit and after. As such, articles were collated to support this stem and to a prime foundation for future events.

**Fig. 1:**
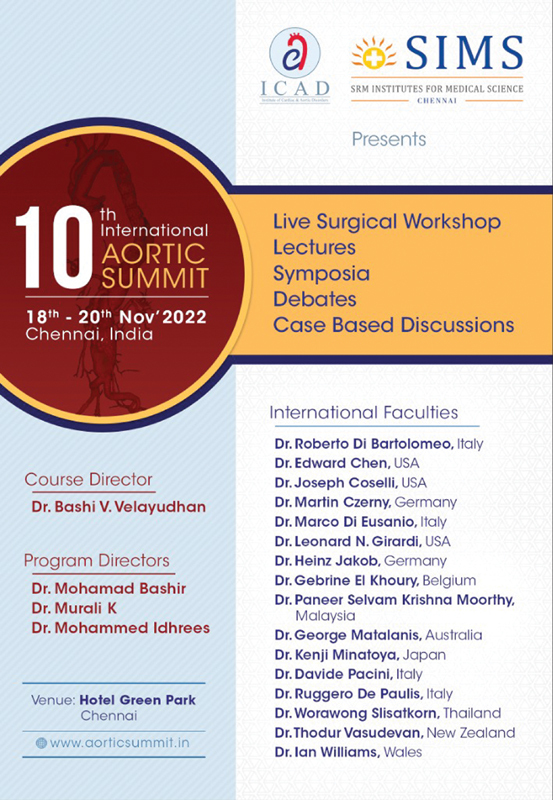
The 10th International Aortic Summit.

With the success of the 9th IAS, we are promising our readers, subscribers, and followers that the 10th IAS will see through live operating workshops performed by esteemed colleagues' debates to induce guidance and appropriate decision-making, and case-based discussions to highlight surgical perspectivity ( Fig. 1 ).

